# Dipotassium tetra­kis­(thio­cyanato-κ*S*)palladate(II)–(2,2′-bipyrimidine-κ^2^ *N* ^1^,*N* ^1′^)bis­(thio­cyanato-κ*S*)palladium(II) (1/2)

**DOI:** 10.1107/S1600536812015383

**Published:** 2012-04-18

**Authors:** Kwang Ha

**Affiliations:** a School of Applied Chemical Engineering, The Research Institute of Catalysis, Chonnam National University, Gwangju 500-757, Republic of Korea

## Abstract

The asymmetric unit of the title compound, K_2_[Pd(NCS)_4_]·2[Pd(NCS)_2_(C_8_H_6_N_4_)], contains two crystallographically independent half-mol­ecules of the anionic Pd^II^ complex, two K^+^ cations and two independent neutral Pd^II^ complexes; an inversion centre is located at the centroid of each anionic complex. In the anionic complexes, each Pd^II^ ion is four-coordinated in an almost regular square-planar environment by four S atoms from four SCN^−^ anions, and the PdS_4_ unit is exactly planar. In the neutral complexes, the Pd^II^ ion has a slightly distorted square-planar coordination environment defined by two pyrimidine N atoms derived from a chelating 2,2′-bipyrimidine ligand and two mutually *cis* S atoms from two SCN^−^ anions. Both 2,2′-bipyrimidine ligands are almost planar [dihedral angle between the rings = 3.98 (16) and 4.57 (17)°] and also chelate to a potassium ion from their other two N atoms. In the crystal, the K^+^ ions inter­act with various S and N atoms of the ligands, forming a three-dimensional polymeric network, in which the shortest K⋯K contacts between the KN_7_S polyhedra are 4.4389 (17) and 4.4966 (18) Å. Intra- and inter­molecular C—H⋯S and C—H⋯N hydrogen bonds are also observed.

## Related literature

For the crystal structure of K_2_[Pd(SCN)_4_], see: Mawby & Pringle (1972 ▶); Ha (2010 ▶).
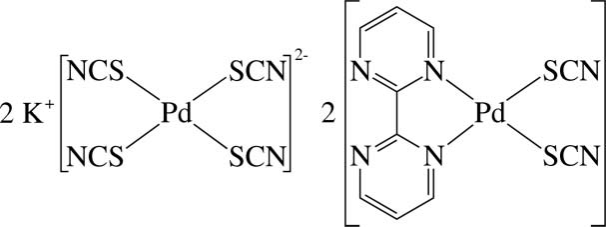


## Experimental

### 

#### Crystal data

K_2_[Pd(NCS)_4_]·2[Pd(NCS)_2_(C_8_H_6_N_4_)]
*M* *_r_* = 1178.38
Monoclinic, 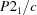

*a* = 15.9625 (7) Å
*b* = 10.9700 (5) Å
*c* = 21.6801 (9) Å
β = 94.104 (1)°
*V* = 3786.6 (3) Å^3^
*Z* = 4
Mo *K*α radiation
μ = 2.12 mm^−1^
*T* = 200 K
0.28 × 0.24 × 0.18 mm

#### Data collection

Bruker SMART 1000 CCD diffractometer
Absorption correction: multi-scan (*SADABS*; Bruker, 2000 ▶) *T*_min_ = 0.854, *T*_max_ = 1.000
22881 measured reflections
7371 independent reflections
5722 reflections with *I* > 2σ(*I*)
*R*_int_ = 0.030

#### Refinement

*R*[*F* ^2^ > 2σ(*F* ^2^)] = 0.030
*wR*(*F* ^2^) = 0.080
*S* = 1.08
7371 reflections
481 parameters
H-atom parameters constrained
Δρ_max_ = 0.65 e Å^−3^
Δρ_min_ = −0.59 e Å^−3^

### 

Data collection: *SMART* (Bruker, 2000 ▶); cell refinement: *SAINT* (Bruker, 2000 ▶); data reduction: *SAINT*; program(s) used to solve structure: *SHELXS97* (Sheldrick, 2008 ▶); program(s) used to refine structure: *SHELXL97* (Sheldrick, 2008 ▶); molecular graphics: *ORTEP-3* (Farrugia, 1997 ▶); software used to prepare material for publication: *SHELXL97*.

## Supplementary Material

Crystal structure: contains datablock(s) global. DOI: 10.1107/S1600536812015383/hb6728sup1.cif

Additional supplementary materials:  crystallographic information; 3D view; checkCIF report

## Figures and Tables

**Table 1:** Selected bond lengths (Å)

Pd1—N1	2.074 (3)
Pd1—N4	2.070 (3)
Pd1—S1	2.2945 (11)
Pd1—S2	2.3077 (11)
Pd2—N7	2.059 (3)
Pd2—N10	2.069 (3)
Pd2—S3^i^	2.2958 (10)
Pd2—S4	2.2829 (11)
Pd3—S5	2.3388 (9)
Pd3—S6	2.3314 (9)
Pd4—S7	2.3157 (10)
Pd4—S8	2.3419 (10)

Symmetry code: (i) 
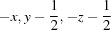
.

**Table 2:** Hydrogen-bond geometry (Å, °)

*D*—H⋯*A*	*D*—H	H⋯*A*	*D*⋯*A*	*D*—H⋯*A*
C1—H1⋯S1	0.95	2.77	3.334 (4)	119
C1—H1⋯N11^ii^	0.95	2.45	3.134 (5)	128
C6—H6⋯S7^iii^	0.95	2.76	3.457 (4)	131
C8—H8⋯S2	0.95	2.77	3.321 (4)	118
C11—H11⋯S3^i^	0.95	2.82	3.371 (4)	118
C11—H11⋯N6^iv^	0.95	2.63	3.315 (5)	130
C13—H13⋯N15^v^	0.95	2.61	3.376 (5)	138
C16—H16⋯S6^ii^	0.95	2.83	3.640 (4)	144
C18—H18⋯S4	0.95	2.71	3.276 (4)	119

Symmetry codes: (i) 
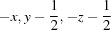
; (ii) 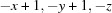; (iii) 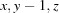; (iv) 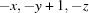; (v) 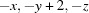.

**Fig. 1.**
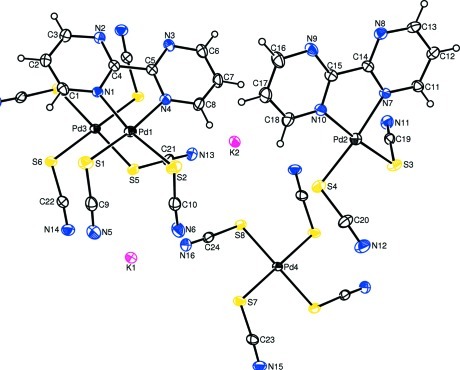
A structure detail of the title compound, with displacement ellipsoids drawn at the 30% probability level for non-H atoms. Unlabelled atoms are generated by the application of the inversion centres.

## supplementary crystallographic information

### Comment

The title compound, K_2_[Pd(SCN)_4_].2[Pd(SCN)_2_(bpym)] (bpym = 2,2'-bipyrimidine), was unexpected obtained from the reaction of Na_2_PdCl_4_ with KSCN and bpym. The asymmetric unit contains two crystallographically independent half-molecules of the anionic Pd^II^ complex [Pd(SCN)_4_]^2-^, two K^+^ cations and two independent neutral Pd^II^ complexes [Pd(SCN)_2_(bpym)]; an inversion centre is located at the centroid of each anionic complex (Fig. 1). The crystal structure of K_2_[Pd(SCN)_4_] has been investigated previously (Mawby & Pringle, 1972; Ha, 2010).

In the anionic complexes, each Pd^II^ ion is four-coordinated in an essentially square-planar environment by four S atoms from four SCN^-^ anions, and the PdS_4_ unit is exactly planar. The two complexes are chemically identical, but slightly different in geometry. The Pd—S bond lengths are roughly equal [Pd—S: 2.3157 (10)–2.3419 (10) Å] (Table 1). The thiocyanato ligands are almost linear displaying S—C—N bond angles of 174.8 (4)°–178.1 (4)°, and the S atoms coordinate to the Pd atom with the nearly tetrahedral Pd—S—C bond angles of 106.57 (13)°–110.44 (13)°.

In the two neutral complexes, each Pd^II^ ion has a slightly distorted square-planar coordination environment defined by two pyrimidine N atoms derived from a chelating bpym ligand and two mutually *cis* S atoms from two SCN^-^ anions. The complexes are fairly different in geometry, because the coordination modes of the anions are significantly different. The two thiocyanato ligands are located on the same side of the PdS_2_N_2_ unit plane in the complex with Pd1, whereas in the other complex with Pd2, the ligands lie on the opposite sides of the PdS_2_N_2_ unit. But, the Pd—N and Pd—S bond lengths are nearly equivalent, respectively [Pd—N = 2.059 (3)–2.074 (3) Å; Pd—S = 2.2829 (11)–2.3077 (11) Å] (Table 1). The bpym ligands are slightly inclined to the least-squares plane of the PdS_2_N_2_ unit [maximum deviation = 0.090 (1) Å], making dihedral angles of 9.55 (9)° in the complex with Pd1 and 7.28 (7)° in the complex with Pd2.

In the crystal structure, the K^+^ ions interact with the various S and N atoms of the ligands with the distances of K···S = 3.6081 (13) Å and 3.6692 (13) Å, and K···N = 2.806 (3)–3.370 (5) Å, forming a three-dimensional polymeric network, and the short K···K contacts are present [4.4389 (17) Å and 4.4966 (18) Å]. Intra- and intramolecular C—H···S and C—H···N hydrogen bonds are also observed (Table 2).

### Experimental

To a solution of Na_2_PdCl_4_ (0.1478 g, 0.502 mmol) in MeOH (30 ml) were added KSCN (0.5358 g, 5.513 mmol) and 2,2'-bipyrimidine (0.0819 g, 0.518 mmol), and refluxed for 3 h. The formed precipitate was separated by filtration and washed with H_2_O and acetone, and dried at 50 °C, to give a pale red powder (0.1095 g). Red blocks were obtained by slow evaporation from a CH_3_CN solution.

### Refinement

H atoms were positioned geometrically and allowed to ride on their respective parent atoms: C—H = 0.95 Å with *U*_iso_(H) = 1.2*U*_eq_(C). The highest peak (0.65 e Å^-3^) and the deepest hole (-0.59 e Å^-3^) in the difference Fourier map are located 0.71 Å and 0.83 Å, respectively, from the Pd1 atom.

### Crystal data

**Table:**

K_2_[Pd(NCS)_4_]·2[Pd(NCS)_2_(C_8_H_6_N_4_)]	*F*(000) = 2288
*M**_r_* = 1178.38	*D*_x_ = 2.067 Mg m^−^^3^
Monoclinic, *P*2_1_/*c*	Mo *K*α radiation, λ = 0.71073 Å
Hall symbol: -P 2ybc	Cell parameters from 5010 reflections
*a* = 15.9625 (7) Å	θ = 2.5–26.0°
*b* = 10.9700 (5) Å	µ = 2.12 mm^−^^1^
*c* = 21.6801 (9) Å	*T* = 200 K
β = 94.104 (1)°	Block, red
*V* = 3786.6 (3) Å^3^	0.28 × 0.24 × 0.18 mm
*Z* = 4	

### Data collection

**Table:**

Bruker SMART 1000 CCD diffractometer	7371 independent reflections
Radiation source: fine-focus sealed tube	5722 reflections with *I* > 2σ(*I*)
Graphite monochromator	*R*_int_ = 0.030
φ and ω scans	θ_max_ = 26.0°, θ_min_ = 1.3°
Absorption correction: multi-scan (*SADABS*; Bruker, 2000)	*h* = −18→19
*T*_min_ = 0.854, *T*_max_ = 1.000	*k* = −12→13
22881 measured reflections	*l* = −25→26

### Refinement

**Table:**

Refinement on *F*^2^	Primary atom site location: structure-invariant direct methods
Least-squares matrix: full	Secondary atom site location: difference Fourier map
*R*[*F*^2^ > 2σ(*F*^2^)] = 0.030	Hydrogen site location: inferred from neighbouring sites
*wR*(*F*^2^) = 0.080	H-atom parameters constrained
*S* = 1.08	*w* = 1/[σ^2^(*F*_o_^2^) + (0.0336*P*)^2^ + 1.0988*P*] where *P* = (*F*_o_^2^ + 2*F*_c_^2^)/3
7371 reflections	(Δ/σ)_max_ = 0.001
481 parameters	Δρ_max_ = 0.65 e Å^−^^3^
0 restraints	Δρ_min_ = −0.59 e Å^−^^3^

### Special details

**Table:**

Geometry. All e.s.d.'s (except the e.s.d. in the dihedral angle between two l.s. planes) are estimated using the full covariance matrix. The cell e.s.d.'s are taken into account individually in the estimation of e.s.d.'s in distances, angles and torsion angles; correlations between e.s.d.'s in cell parameters are only used when they are defined by crystal symmetry. An approximate (isotropic) treatment of cell e.s.d.'s is used for estimating e.s.d.'s involving l.s. planes.
Refinement. Refinement of *F*^2^ against ALL reflections. The weighted *R*-factor *wR* and goodness of fit *S* are based on *F*^2^, conventional *R*-factors *R* are based on *F*, with *F* set to zero for negative *F*^2^. The threshold expression of *F*^2^ > σ(*F*^2^) is used only for calculating *R*-factors(gt) *etc*. and is not relevant to the choice of reflections for refinement. *R*-factors based on *F*^2^ are statistically about twice as large as those based on *F*, and *R*- factors based on ALL data will be even larger.

### Fractional atomic coordinates and isotropic or equivalent isotropic displacement parameters (Å^2^)

**Table:**

	*x*	*y*	*z*	*U*_iso_*/*U*_eq_	
Pd1	0.482313 (18)	0.51068 (2)	0.188694 (12)	0.03065 (9)	
Pd2	−0.014348 (18)	−0.00967 (2)	−0.182524 (12)	0.02913 (8)	
Pd3	0.5000	0.5000	0.0000	0.02538 (10)	
Pd4	0.0000	1.0000	0.0000	0.02620 (10)	
K1	0.42675 (5)	0.96456 (8)	0.08137 (4)	0.0397 (2)	
K2	0.09465 (6)	0.52691 (8)	−0.07076 (4)	0.0426 (2)	
S1	0.59836 (7)	0.62463 (10)	0.21959 (5)	0.0483 (3)	
S2	0.39550 (7)	0.62960 (10)	0.24449 (5)	0.0498 (3)	
S3	0.13902 (6)	0.40429 (9)	−0.28031 (5)	0.0412 (2)	
S4	0.05796 (7)	−0.17773 (10)	−0.21091 (5)	0.0515 (3)	
S5	0.41359 (6)	0.64489 (9)	0.04273 (5)	0.0376 (2)	
S6	0.61598 (6)	0.62728 (9)	0.01879 (5)	0.0396 (2)	
S7	0.11212 (6)	1.12966 (9)	0.02391 (5)	0.0448 (3)	
S8	0.09601 (6)	0.84561 (9)	−0.01933 (5)	0.0405 (2)	
N1	0.55052 (17)	0.3754 (2)	0.14802 (12)	0.0263 (6)	
N2	0.53434 (18)	0.1777 (3)	0.10408 (12)	0.0312 (7)	
N3	0.36456 (18)	0.1932 (3)	0.11846 (13)	0.0330 (7)	
N4	0.38671 (18)	0.3913 (3)	0.16051 (12)	0.0302 (7)	
N5	0.5592 (2)	0.8652 (3)	0.18054 (17)	0.0528 (9)	
N6	0.3527 (2)	0.8499 (4)	0.18235 (19)	0.0607 (11)	
N7	−0.06619 (17)	0.1506 (3)	−0.15385 (12)	0.0280 (6)	
N8	−0.02642 (18)	0.3475 (3)	−0.11603 (13)	0.0347 (7)	
N9	0.13917 (19)	0.2811 (3)	−0.12042 (14)	0.0381 (8)	
N10	0.09353 (18)	0.0838 (3)	−0.15250 (13)	0.0320 (7)	
N11	0.17991 (19)	0.5602 (3)	−0.17926 (16)	0.0460 (9)	
N12	−0.0430 (3)	−0.3800 (4)	−0.1863 (2)	0.0694 (12)	
N13	0.2462 (2)	0.5816 (3)	0.00536 (17)	0.0509 (9)	
N14	0.5701 (2)	0.8743 (3)	0.02917 (15)	0.0427 (8)	
N15	0.0555 (2)	1.3726 (3)	0.03105 (16)	0.0469 (9)	
N16	0.2599 (2)	0.9214 (3)	0.02070 (17)	0.0516 (9)	
C1	0.6330 (2)	0.3698 (4)	0.14158 (16)	0.0353 (9)	
H1	0.6675	0.4368	0.1549	0.042*	
C2	0.6693 (2)	0.2698 (3)	0.11627 (16)	0.0355 (9)	
H2	0.7280	0.2661	0.1118	0.043*	
C3	0.6171 (2)	0.1759 (4)	0.09784 (16)	0.0372 (9)	
H3	0.6407	0.1062	0.0798	0.045*	
C4	0.5048 (2)	0.2778 (3)	0.12903 (14)	0.0268 (8)	
C5	0.4131 (2)	0.2861 (3)	0.13633 (14)	0.0272 (8)	
C6	0.2825 (2)	0.2081 (4)	0.12340 (17)	0.0402 (10)	
H6	0.2458	0.1427	0.1117	0.048*	
C7	0.2488 (2)	0.3139 (4)	0.14461 (18)	0.0441 (10)	
H7	0.1899	0.3243	0.1459	0.053*	
C8	0.3038 (2)	0.4030 (4)	0.16362 (17)	0.0398 (9)	
H8	0.2825	0.4764	0.1797	0.048*	
C9	0.5728 (2)	0.7657 (4)	0.19553 (18)	0.0411 (10)	
C10	0.3729 (2)	0.7573 (4)	0.20423 (18)	0.0405 (10)	
C11	−0.1472 (2)	0.1804 (3)	−0.15213 (16)	0.0329 (8)	
H11	−0.1894	0.1226	−0.1647	0.039*	
C12	−0.1702 (2)	0.2942 (3)	−0.13225 (16)	0.0370 (9)	
H12	−0.2277	0.3157	−0.1307	0.044*	
C13	−0.1075 (2)	0.3756 (3)	−0.11476 (17)	0.0378 (9)	
H13	−0.1225	0.4546	−0.1013	0.045*	
C14	−0.0094 (2)	0.2372 (3)	−0.13530 (15)	0.0293 (8)	
C15	0.0802 (2)	0.2000 (3)	−0.13587 (15)	0.0309 (8)	
C16	0.2179 (2)	0.2413 (4)	−0.12183 (19)	0.0487 (11)	
H16	0.2624	0.2973	−0.1126	0.058*	
C17	0.2379 (3)	0.1234 (4)	−0.1360 (2)	0.0517 (11)	
H17	0.2946	0.0969	−0.1352	0.062*	
C18	0.1730 (2)	0.0461 (4)	−0.15129 (19)	0.0445 (10)	
H18	0.1846	−0.0363	−0.1613	0.053*	
C19	0.1617 (2)	0.4984 (3)	−0.22118 (18)	0.0342 (9)	
C20	−0.0035 (3)	−0.2959 (4)	−0.19531 (18)	0.0452 (10)	
C21	0.3143 (2)	0.6055 (3)	0.02093 (17)	0.0379 (9)	
C22	0.5851 (2)	0.7726 (4)	0.02536 (16)	0.0328 (8)	
C23	0.0761 (2)	1.2729 (4)	0.02831 (17)	0.0362 (9)	
C24	0.1932 (2)	0.8932 (3)	0.00439 (17)	0.0373 (9)	

### Atomic displacement parameters (Å^2^)

**Table:**

	*U*^11^	*U*^22^	*U*^33^	*U*^12^	*U*^13^	*U*^23^
Pd1	0.03478 (17)	0.02747 (16)	0.02909 (15)	−0.00023 (12)	−0.00199 (12)	−0.00338 (12)
Pd2	0.03272 (16)	0.02533 (16)	0.02903 (15)	−0.00017 (12)	0.00016 (12)	−0.00167 (12)
Pd3	0.0290 (2)	0.0225 (2)	0.02406 (19)	0.00083 (15)	−0.00188 (15)	0.00014 (15)
Pd4	0.0283 (2)	0.0216 (2)	0.0280 (2)	−0.00027 (15)	−0.00313 (16)	0.00051 (16)
K1	0.0359 (5)	0.0320 (5)	0.0517 (5)	−0.0038 (4)	0.0055 (4)	−0.0031 (4)
K2	0.0389 (5)	0.0381 (5)	0.0512 (5)	−0.0072 (4)	0.0072 (4)	−0.0074 (4)
S1	0.0455 (6)	0.0389 (6)	0.0580 (7)	−0.0035 (5)	−0.0140 (5)	−0.0123 (5)
S2	0.0631 (7)	0.0439 (6)	0.0430 (6)	0.0079 (5)	0.0090 (5)	−0.0079 (5)
S3	0.0449 (6)	0.0334 (6)	0.0442 (6)	0.0121 (4)	−0.0041 (5)	−0.0023 (5)
S4	0.0507 (7)	0.0383 (6)	0.0668 (7)	0.0042 (5)	0.0134 (6)	−0.0161 (6)
S5	0.0363 (5)	0.0318 (5)	0.0449 (5)	0.0030 (4)	0.0035 (4)	−0.0079 (4)
S6	0.0338 (5)	0.0279 (5)	0.0558 (6)	−0.0010 (4)	−0.0051 (5)	−0.0041 (5)
S7	0.0336 (6)	0.0276 (5)	0.0713 (7)	−0.0023 (4)	−0.0106 (5)	−0.0040 (5)
S8	0.0338 (5)	0.0302 (5)	0.0568 (6)	0.0030 (4)	−0.0014 (5)	−0.0082 (5)
N1	0.0283 (16)	0.0246 (16)	0.0259 (14)	−0.0013 (12)	0.0002 (12)	−0.0035 (12)
N2	0.0306 (17)	0.0335 (18)	0.0294 (15)	−0.0005 (13)	0.0008 (13)	−0.0028 (14)
N3	0.0296 (17)	0.0369 (18)	0.0322 (16)	−0.0059 (14)	−0.0009 (13)	−0.0015 (14)
N4	0.0325 (17)	0.0305 (17)	0.0273 (15)	0.0023 (13)	0.0005 (13)	0.0041 (13)
N5	0.050 (2)	0.047 (2)	0.061 (2)	−0.0081 (19)	0.0080 (18)	−0.003 (2)
N6	0.035 (2)	0.054 (3)	0.092 (3)	−0.0003 (18)	−0.0020 (19)	0.028 (2)
N7	0.0288 (16)	0.0247 (16)	0.0302 (15)	−0.0014 (12)	−0.0001 (13)	0.0008 (13)
N8	0.0339 (18)	0.0308 (18)	0.0386 (17)	−0.0005 (14)	−0.0028 (14)	−0.0057 (14)
N9	0.0316 (18)	0.0382 (19)	0.0435 (18)	−0.0095 (15)	−0.0049 (14)	0.0035 (15)
N10	0.0316 (17)	0.0307 (18)	0.0333 (16)	−0.0012 (13)	0.0003 (13)	0.0021 (14)
N11	0.0307 (19)	0.051 (2)	0.055 (2)	0.0003 (16)	−0.0048 (16)	−0.0065 (19)
N12	0.079 (3)	0.041 (2)	0.092 (3)	0.017 (2)	0.035 (2)	0.011 (2)
N13	0.036 (2)	0.046 (2)	0.072 (2)	0.0018 (17)	0.0105 (18)	−0.0076 (19)
N14	0.046 (2)	0.034 (2)	0.0476 (19)	−0.0019 (16)	0.0009 (16)	−0.0057 (16)
N15	0.049 (2)	0.030 (2)	0.061 (2)	−0.0015 (16)	−0.0027 (17)	−0.0088 (17)
N16	0.032 (2)	0.045 (2)	0.078 (3)	0.0001 (16)	0.0049 (18)	−0.001 (2)
C1	0.031 (2)	0.040 (2)	0.034 (2)	−0.0060 (17)	−0.0021 (16)	0.0059 (17)
C2	0.030 (2)	0.042 (2)	0.034 (2)	0.0014 (17)	0.0050 (16)	0.0010 (18)
C3	0.033 (2)	0.042 (2)	0.037 (2)	0.0028 (18)	0.0042 (17)	−0.0032 (18)
C4	0.0296 (19)	0.030 (2)	0.0202 (17)	−0.0024 (15)	−0.0009 (14)	0.0026 (15)
C5	0.0303 (19)	0.030 (2)	0.0211 (17)	−0.0024 (16)	−0.0013 (14)	0.0025 (15)
C6	0.031 (2)	0.047 (3)	0.042 (2)	−0.0082 (18)	−0.0003 (17)	0.0030 (19)
C7	0.021 (2)	0.053 (3)	0.058 (3)	−0.0011 (18)	0.0021 (18)	0.006 (2)
C8	0.030 (2)	0.042 (2)	0.048 (2)	0.0082 (18)	0.0075 (18)	0.005 (2)
C9	0.034 (2)	0.050 (3)	0.040 (2)	−0.0090 (19)	0.0030 (17)	−0.014 (2)
C10	0.035 (2)	0.046 (3)	0.040 (2)	−0.0071 (19)	0.0047 (18)	−0.013 (2)
C11	0.031 (2)	0.031 (2)	0.037 (2)	−0.0038 (16)	0.0000 (16)	−0.0005 (17)
C12	0.035 (2)	0.036 (2)	0.039 (2)	0.0056 (17)	0.0010 (17)	−0.0055 (18)
C13	0.043 (2)	0.027 (2)	0.043 (2)	0.0051 (17)	−0.0013 (18)	−0.0066 (18)
C14	0.031 (2)	0.028 (2)	0.0278 (18)	−0.0038 (16)	0.0001 (15)	0.0011 (16)
C15	0.033 (2)	0.031 (2)	0.0280 (19)	−0.0020 (16)	−0.0033 (15)	0.0037 (16)
C16	0.032 (2)	0.058 (3)	0.055 (3)	−0.012 (2)	−0.0032 (19)	0.008 (2)
C17	0.029 (2)	0.054 (3)	0.071 (3)	0.002 (2)	0.000 (2)	0.009 (2)
C18	0.030 (2)	0.044 (2)	0.059 (3)	0.0055 (18)	0.0024 (19)	−0.002 (2)
C19	0.0235 (19)	0.035 (2)	0.044 (2)	0.0031 (16)	0.0012 (17)	0.0090 (19)
C20	0.059 (3)	0.039 (3)	0.039 (2)	0.017 (2)	0.013 (2)	0.001 (2)
C21	0.037 (2)	0.034 (2)	0.044 (2)	0.0084 (18)	0.0138 (19)	0.0008 (19)
C22	0.034 (2)	0.036 (2)	0.0283 (19)	−0.0049 (17)	−0.0007 (16)	−0.0011 (17)
C23	0.038 (2)	0.032 (2)	0.037 (2)	−0.0063 (17)	−0.0045 (17)	−0.0057 (18)
C24	0.040 (2)	0.031 (2)	0.042 (2)	0.0073 (18)	0.0102 (18)	0.0034 (18)

### Geometric parameters (Å, º)

**Table:**

Pd1—N1	2.074 (3)	N3—C5	1.321 (4)
Pd1—N4	2.070 (3)	N3—C6	1.331 (4)
Pd1—S1	2.2945 (11)	N3—K1^vi^	2.835 (3)
Pd1—S2	2.3077 (11)	N4—C8	1.336 (4)
Pd2—N7	2.059 (3)	N4—C5	1.348 (4)
Pd2—N10	2.069 (3)	N5—C9	1.155 (5)
Pd2—S3^i^	2.2958 (10)	N6—C10	1.158 (5)
Pd2—S4	2.2829 (11)	N7—C11	1.337 (4)
Pd3—S5^ii^	2.3388 (9)	N7—C14	1.354 (4)
Pd3—S5	2.3388 (9)	N8—C14	1.315 (4)
Pd3—S6^ii^	2.3314 (9)	N8—C13	1.333 (4)
Pd3—S6	2.3314 (9)	N9—C15	1.321 (4)
Pd4—S7^iii^	2.3157 (10)	N9—C16	1.333 (5)
Pd4—S7	2.3157 (10)	N10—C18	1.333 (5)
Pd4—S8^iii^	2.3419 (10)	N10—C15	1.346 (4)
Pd4—S8	2.3419 (10)	N11—C19	1.154 (5)
K1—N14	2.806 (3)	N12—C20	1.142 (5)
K1—N3^iv^	2.835 (3)	N12—K2^vi^	3.370 (5)
K1—N6	2.853 (4)	N13—C21	1.146 (5)
K1—N2^iv^	2.922 (3)	N14—C22	1.145 (5)
K1—N16	2.923 (4)	N14—K1^v^	2.981 (3)
K1—N14^v^	2.981 (3)	N15—C23	1.144 (5)
K1—N5	3.103 (4)	N15—K2^iii^	2.827 (4)
K1—S5	3.6081 (13)	N15—K2^iv^	2.886 (4)
K1—K1^v^	4.4389 (17)	N16—C24	1.141 (5)
K2—N11	2.824 (4)	C1—C2	1.373 (5)
K2—N15^iii^	2.827 (4)	C1—H1	0.9500
K2—N8	2.881 (3)	C2—C3	1.366 (5)
K2—N15^vi^	2.886 (4)	C2—H2	0.9500
K2—N13	2.892 (4)	C3—H3	0.9500
K2—N9	3.008 (3)	C4—C5	1.486 (5)
K2—N12^iv^	3.370 (5)	C6—C7	1.373 (5)
K2—S8	3.6692 (13)	C6—H6	0.9500
K2—K2^vii^	4.4966 (18)	C7—C8	1.358 (5)
S1—C9	1.674 (5)	C7—H7	0.9500
S2—C10	1.676 (5)	C8—H8	0.9500
S3—C19	1.666 (4)	C11—C12	1.379 (5)
S3—Pd2^viii^	2.2958 (10)	C11—H11	0.9500
S4—C20	1.675 (5)	C12—C13	1.375 (5)
S5—C21	1.677 (4)	C12—H12	0.9500
S6—C22	1.678 (4)	C13—H13	0.9500
S7—C23	1.679 (4)	C14—C15	1.487 (5)
S8—C24	1.683 (4)	C16—C17	1.372 (6)
N1—C1	1.336 (4)	C16—H16	0.9500
N1—C4	1.344 (4)	C17—C18	1.361 (6)
N2—C4	1.325 (4)	C17—H17	0.9500
N2—C3	1.338 (4)	C18—H18	0.9500
N2—K1^vi^	2.922 (3)		
			
N4—Pd1—N1	79.65 (11)	C10—S2—Pd1	108.31 (14)
N4—Pd1—S1	173.55 (8)	C19—S3—Pd2^viii^	98.67 (13)
N1—Pd1—S1	94.39 (8)	C20—S4—Pd2	104.84 (14)
N4—Pd1—S2	93.13 (8)	C21—S5—Pd3	106.57 (13)
N1—Pd1—S2	168.65 (8)	C21—S5—K1	110.74 (13)
S1—Pd1—S2	92.38 (4)	Pd3—S5—K1	136.58 (4)
N7—Pd2—N10	79.94 (11)	C22—S6—Pd3	110.44 (13)
N7—Pd2—S4	173.34 (8)	C23—S7—Pd4	108.97 (13)
N10—Pd2—S4	93.40 (9)	C24—S8—Pd4	108.78 (13)
N7—Pd2—S3^i^	95.74 (8)	C24—S8—K2	111.96 (13)
N10—Pd2—S3^i^	174.43 (8)	Pd4—S8—K2	138.91 (4)
S4—Pd2—S3^i^	90.90 (4)	C1—N1—C4	116.7 (3)
S6^ii^—Pd3—S6	180.00 (5)	C1—N1—Pd1	128.9 (2)
S6^ii^—Pd3—S5^ii^	90.42 (3)	C4—N1—Pd1	114.2 (2)
S6—Pd3—S5^ii^	89.58 (3)	C4—N2—C3	115.8 (3)
S6^ii^—Pd3—S5	89.58 (3)	C4—N2—K1^vi^	120.7 (2)
S6—Pd3—S5	90.42 (3)	C3—N2—K1^vi^	123.0 (2)
S5^ii^—Pd3—S5	180.00 (6)	C5—N3—C6	116.2 (3)
S7^iii^—Pd4—S7	180.00 (6)	C5—N3—K1^vi^	123.7 (2)
S7^iii^—Pd4—S8^iii^	88.80 (3)	C6—N3—K1^vi^	119.8 (2)
S7—Pd4—S8^iii^	91.20 (3)	C8—N4—C5	116.1 (3)
S7^iii^—Pd4—S8	91.20 (3)	C8—N4—Pd1	129.5 (3)
S7—Pd4—S8	88.80 (4)	C5—N4—Pd1	114.3 (2)
S8^iii^—Pd4—S8	180.00 (5)	C9—N5—K1	129.2 (3)
N14—K1—N3^iv^	137.64 (9)	C10—N6—K1	125.6 (3)
N14—K1—N6	123.28 (11)	C11—N7—C14	116.7 (3)
N3^iv^—K1—N6	89.98 (10)	C11—N7—Pd2	128.9 (2)
N14—K1—N2^iv^	82.17 (9)	C14—N7—Pd2	114.4 (2)
N3^iv^—K1—N2^iv^	57.16 (8)	C14—N8—C13	116.3 (3)
N6—K1—N2^iv^	119.60 (10)	C14—N8—K2	126.0 (2)
N14—K1—N16	120.30 (10)	C13—N8—K2	117.6 (2)
N3^iv^—K1—N16	86.83 (9)	C15—N9—C16	115.5 (3)
N6—K1—N16	81.96 (10)	C15—N9—K2	120.8 (2)
N2^iv^—K1—N16	135.31 (10)	C16—N9—K2	123.3 (3)
N14—K1—N14^v^	79.86 (10)	C18—N10—C15	117.2 (3)
N3^iv^—K1—N14^v^	74.32 (9)	C18—N10—Pd2	128.5 (3)
N6—K1—N14^v^	155.48 (10)	C15—N10—Pd2	114.2 (2)
N2^iv^—K1—N14^v^	67.52 (8)	C19—N11—K2	118.1 (3)
N16—K1—N14^v^	78.53 (10)	C20—N12—K2^vi^	91.9 (3)
N14—K1—N5	67.69 (10)	C21—N13—K2	162.4 (3)
N3^iv^—K1—N5	110.45 (9)	C22—N14—K1	123.6 (3)
N6—K1—N5	67.21 (10)	C22—N14—K1^v^	120.3 (3)
N2^iv^—K1—N5	78.54 (9)	K1—N14—K1^v^	100.14 (10)
N16—K1—N5	143.95 (10)	C23—N15—K2^iii^	129.9 (3)
N14^v^—K1—N5	135.69 (9)	C23—N15—K2^iv^	116.2 (3)
N14—K1—S5	66.36 (7)	K2^iii^—N15—K2^iv^	103.82 (11)
N3^iv^—K1—S5	155.51 (7)	C24—N16—K1	169.6 (3)
N6—K1—S5	74.30 (9)	N1—C1—C2	121.7 (3)
N2^iv^—K1—S5	147.20 (7)	N1—C1—H1	119.2
N16—K1—S5	72.67 (7)	C2—C1—H1	119.2
N14^v^—K1—S5	113.18 (7)	C3—C2—C1	117.0 (3)
N5—K1—S5	81.08 (7)	C3—C2—H2	121.5
N14—K1—K1^v^	41.39 (7)	C1—C2—H2	121.5
N3^iv^—K1—K1^v^	106.59 (7)	N2—C3—C2	123.0 (4)
N6—K1—K1^v^	163.30 (9)	N2—C3—H3	118.5
N2^iv^—K1—K1^v^	69.94 (6)	C2—C3—H3	118.5
N16—K1—K1^v^	100.68 (8)	N2—C4—N1	125.7 (3)
N14^v^—K1—K1^v^	38.47 (7)	N2—C4—C5	118.4 (3)
N5—K1—K1^v^	103.93 (7)	N1—C4—C5	115.8 (3)
S5—K1—K1^v^	90.63 (3)	N3—C5—N4	125.5 (3)
N11—K2—N15^iii^	132.28 (10)	N3—C5—C4	118.9 (3)
N11—K2—N8	98.97 (9)	N4—C5—C4	115.6 (3)
N15^iii^—K2—N8	78.92 (9)	N3—C6—C7	122.8 (4)
N11—K2—N15^vi^	148.93 (10)	N3—C6—H6	118.6
N15^iii^—K2—N15^vi^	76.18 (11)	C7—C6—H6	118.6
N8—K2—N15^vi^	71.51 (9)	C8—C7—C6	116.7 (4)
N11—K2—N13	90.88 (10)	C8—C7—H7	121.6
N15^iii^—K2—N13	115.96 (10)	C6—C7—H7	121.6
N8—K2—N13	148.14 (10)	N4—C8—C7	122.5 (4)
N15^vi^—K2—N13	84.46 (10)	N4—C8—H8	118.8
N11—K2—N9	71.18 (9)	C7—C8—H8	118.8
N15^iii^—K2—N9	133.30 (9)	N5—C9—S1	176.3 (4)
N8—K2—N9	55.62 (8)	N6—C10—S2	172.0 (4)
N15^vi^—K2—N9	79.48 (9)	N7—C11—C12	120.6 (3)
N13—K2—N9	100.43 (9)	N7—C11—H11	119.7
N11—K2—N12^iv^	70.69 (9)	C12—C11—H11	119.7
N15^iii^—K2—N12^iv^	65.57 (10)	C13—C12—C11	118.0 (3)
N8—K2—N12^iv^	64.42 (10)	C13—C12—H12	121.0
N15^vi^—K2—N12^iv^	125.51 (10)	C11—C12—H12	121.0
N13—K2—N12^iv^	146.66 (11)	N8—C13—C12	122.2 (3)
N9—K2—N12^iv^	99.44 (9)	N8—C13—H13	118.9
N11—K2—C19	16.81 (8)	C12—C13—H13	118.9
N15^iii^—K2—C19	129.62 (9)	N8—C14—N7	126.1 (3)
N8—K2—C19	82.22 (9)	N8—C14—C15	118.4 (3)
N15^vi^—K2—C19	138.92 (9)	N7—C14—C15	115.4 (3)
N13—K2—C19	104.47 (9)	N9—C15—N10	125.6 (3)
N9—K2—C19	59.54 (8)	N9—C15—C14	118.8 (3)
N12^iv^—K2—C19	64.12 (9)	N10—C15—C14	115.6 (3)
N11—K2—S8	97.90 (8)	N9—C16—C17	123.2 (4)
N15^iii^—K2—S8	61.49 (7)	N9—C16—H16	118.4
N8—K2—S8	138.05 (7)	C17—C16—H16	118.4
N15^vi^—K2—S8	108.87 (7)	C18—C17—C16	117.2 (4)
N13—K2—S8	69.04 (7)	C18—C17—H17	121.4
N9—K2—S8	165.17 (7)	C16—C17—H17	121.4
N12^iv^—K2—S8	85.78 (7)	N10—C18—C17	121.2 (4)
C19—K2—S8	111.77 (7)	N10—C18—H18	119.4
N11—K2—K2^vii^	166.57 (8)	C17—C18—H18	119.4
N15^iii^—K2—K2^vii^	38.55 (7)	N11—C19—S3	177.2 (4)
N8—K2—K2^vii^	71.07 (6)	N11—C19—K2	45.1 (2)
N15^vi^—K2—K2^vii^	37.63 (7)	S3—C19—K2	134.80 (17)
N13—K2—K2^vii^	102.31 (8)	N12—C20—S4	176.7 (4)
N9—K2—K2^vii^	108.30 (7)	N13—C21—S5	178.1 (4)
N12^iv^—K2—K2^vii^	96.47 (7)	N14—C22—S6	174.8 (4)
C19—K2—K2^vii^	152.22 (7)	N15—C23—S7	176.6 (4)
S8—K2—K2^vii^	84.69 (3)	N16—C24—S8	177.7 (4)
C9—S1—Pd1	103.83 (14)		
			
N1—Pd1—S1—C9	130.92 (16)	S4—Pd2—N10—C15	173.9 (2)
S2—Pd1—S1—C9	−58.20 (15)	N15^iii^—K2—N11—C19	−88.3 (3)
N4—Pd1—S2—C10	−104.11 (16)	N8—K2—N11—C19	−5.2 (3)
N1—Pd1—S2—C10	−154.2 (4)	N15^vi^—K2—N11—C19	63.7 (4)
S1—Pd1—S2—C10	79.23 (14)	N13—K2—N11—C19	144.4 (3)
N10—Pd2—S4—C20	145.08 (17)	N9—K2—N11—C19	43.7 (3)
S3^i^—Pd2—S4—C20	−38.46 (16)	N12^iv^—K2—N11—C19	−64.0 (3)
S6^ii^—Pd3—S5—C21	−15.44 (14)	S8—K2—N11—C19	−146.6 (3)
S6—Pd3—S5—C21	164.56 (14)	K2^vii^—K2—N11—C19	−46.4 (6)
S6^ii^—Pd3—S5—K1	−163.85 (6)	N11—K2—N13—C21	−1.9 (10)
S6—Pd3—S5—K1	16.15 (6)	N15^iii^—K2—N13—C21	−141.0 (10)
N14—K1—S5—C21	−147.04 (16)	N8—K2—N13—C21	106.8 (10)
N3^iv^—K1—S5—C21	22.9 (2)	N15^vi^—K2—N13—C21	147.3 (10)
N6—K1—S5—C21	74.78 (16)	N9—K2—N13—C21	69.1 (10)
N2^iv^—K1—S5—C21	−164.47 (17)	N12^iv^—K2—N13—C21	−56.5 (11)
N16—K1—S5—C21	−11.52 (16)	C19—K2—N13—C21	8.1 (11)
N14^v^—K1—S5—C21	−80.41 (16)	S8—K2—N13—C21	−100.0 (10)
N5—K1—S5—C21	143.48 (16)	K2^vii^—K2—N13—C21	−179.3 (10)
K1^v^—K1—S5—C21	−112.52 (14)	N3^iv^—K1—N14—C22	170.0 (3)
N14—K1—S5—Pd3	0.49 (9)	N6—K1—N14—C22	34.0 (4)
N3^iv^—K1—S5—Pd3	170.47 (15)	N2^iv^—K1—N14—C22	154.4 (3)
N6—K1—S5—Pd3	−137.68 (9)	N16—K1—N14—C22	−67.0 (4)
N2^iv^—K1—S5—Pd3	−16.93 (14)	N14^v^—K1—N14—C22	−137.2 (4)
N16—K1—S5—Pd3	136.02 (9)	N5—K1—N14—C22	73.6 (3)
N14^v^—K1—S5—Pd3	67.13 (9)	S5—K1—N14—C22	−16.2 (3)
N5—K1—S5—Pd3	−68.98 (9)	K1^v^—K1—N14—C22	−137.2 (4)
K1^v^—K1—S5—Pd3	35.02 (6)	N3^iv^—K1—N14—K1^v^	−52.87 (16)
S5^ii^—Pd3—S6—C22	157.74 (14)	N6—K1—N14—K1^v^	171.15 (11)
S5—Pd3—S6—C22	−22.26 (14)	N2^iv^—K1—N14—K1^v^	−68.44 (9)
S8—Pd4—S7—C23	168.18 (15)	N16—K1—N14—K1^v^	70.22 (13)
S7^iii^—Pd4—S8—C24	−170.46 (14)	N14^v^—K1—N14—K1^v^	0.0
S7—Pd4—S8—C24	9.54 (14)	N5—K1—N14—K1^v^	−149.21 (12)
S7^iii^—Pd4—S8—K2	1.77 (7)	S5—K1—N14—K1^v^	120.99 (9)
S7—Pd4—S8—K2	−178.23 (7)	N14—K1—N16—C24	110.1 (18)
N11—K2—S8—C24	−69.23 (16)	N3^iv^—K1—N16—C24	−104.3 (18)
N15^iii^—K2—S8—C24	156.54 (17)	N6—K1—N16—C24	−13.9 (18)
N8—K2—S8—C24	177.82 (17)	N2^iv^—K1—N16—C24	−138.4 (17)
N15^vi^—K2—S8—C24	94.77 (16)	N5—K1—N16—C24	16.9 (19)
N13—K2—S8—C24	18.67 (16)	S5—K1—N16—C24	62.1 (18)
N9—K2—S8—C24	−27.7 (3)	K1^v^—K1—N16—C24	149.4 (18)
N12^iv^—K2—S8—C24	−139.05 (16)	C4—N1—C1—C2	0.6 (5)
C19—K2—S8—C24	−79.09 (16)	Pd1—N1—C1—C2	176.4 (2)
K2^vii^—K2—S8—C24	124.04 (14)	N1—C1—C2—C3	0.0 (5)
N11—K2—S8—Pd4	118.71 (9)	C4—N2—C3—C2	0.6 (5)
N15^iii^—K2—S8—Pd4	−15.52 (10)	K1^vi^—N2—C3—C2	−170.9 (3)
N8—K2—S8—Pd4	5.76 (13)	C1—C2—C3—N2	−0.7 (5)
N15^vi^—K2—S8—Pd4	−77.29 (10)	C3—N2—C4—N1	0.1 (5)
N13—K2—S8—Pd4	−153.39 (10)	K1^vi^—N2—C4—N1	171.9 (2)
N9—K2—S8—Pd4	160.2 (2)	C3—N2—C4—C5	179.1 (3)
N12^iv^—K2—S8—Pd4	48.89 (9)	K1^vi^—N2—C4—C5	−9.1 (4)
C19—K2—S8—Pd4	108.84 (9)	C1—N1—C4—N2	−0.7 (5)
K2^vii^—K2—S8—Pd4	−48.03 (7)	Pd1—N1—C4—N2	−177.1 (3)
N4—Pd1—N1—C1	179.1 (3)	C1—N1—C4—C5	−179.7 (3)
S1—Pd1—N1—C1	−3.3 (3)	Pd1—N1—C4—C5	3.9 (4)
S2—Pd1—N1—C1	−129.7 (4)	C6—N3—C5—N4	2.2 (5)
N4—Pd1—N1—C4	−5.0 (2)	K1^vi^—N3—C5—N4	−172.4 (2)
S1—Pd1—N1—C4	172.5 (2)	C6—N3—C5—C4	−176.9 (3)
S2—Pd1—N1—C4	46.1 (5)	K1^vi^—N3—C5—C4	8.5 (4)
N1—Pd1—N4—C8	−175.6 (3)	C8—N4—C5—N3	−3.3 (5)
S2—Pd1—N4—C8	13.3 (3)	Pd1—N4—C5—N3	175.9 (3)
N1—Pd1—N4—C5	5.4 (2)	C8—N4—C5—C4	175.9 (3)
S2—Pd1—N4—C5	−165.8 (2)	Pd1—N4—C5—C4	−5.0 (3)
N14—K1—N5—C9	−80.2 (4)	N2—C4—C5—N3	0.8 (5)
N3^iv^—K1—N5—C9	145.5 (4)	N1—C4—C5—N3	179.9 (3)
N6—K1—N5—C9	64.5 (4)	N2—C4—C5—N4	−178.4 (3)
N2^iv^—K1—N5—C9	−166.3 (4)	N1—C4—C5—N4	0.7 (4)
N16—K1—N5—C9	31.1 (5)	C5—N3—C6—C7	1.2 (5)
N14^v^—K1—N5—C9	−126.3 (4)	K1^vi^—N3—C6—C7	176.0 (3)
S5—K1—N5—C9	−12.2 (4)	N3—C6—C7—C8	−3.1 (6)
K1^v^—K1—N5—C9	−100.6 (4)	C5—N4—C8—C7	1.0 (5)
N14—K1—N6—C10	−6.0 (4)	Pd1—N4—C8—C7	−178.0 (3)
N3^iv^—K1—N6—C10	−158.1 (4)	C6—C7—C8—N4	1.9 (6)
N2^iv^—K1—N6—C10	−106.7 (4)	C14—N7—C11—C12	0.0 (5)
N16—K1—N6—C10	115.1 (4)	Pd2—N7—C11—C12	−178.9 (3)
N14^v^—K1—N6—C10	152.6 (3)	N7—C11—C12—C13	0.4 (5)
N5—K1—N6—C10	−45.8 (4)	C14—N8—C13—C12	0.3 (5)
S5—K1—N6—C10	41.0 (4)	K2—N8—C13—C12	−176.5 (3)
K1^v^—K1—N6—C10	14.7 (6)	C11—C12—C13—N8	−0.6 (6)
N10—Pd2—N7—C11	−176.4 (3)	C13—N8—C14—N7	0.1 (5)
S3^i^—Pd2—N7—C11	7.2 (3)	K2—N8—C14—N7	176.7 (2)
N10—Pd2—N7—C14	4.7 (2)	C13—N8—C14—C15	−178.3 (3)
S3^i^—Pd2—N7—C14	−171.7 (2)	K2—N8—C14—C15	−1.7 (4)
N11—K2—N8—C14	63.2 (3)	C11—N7—C14—N8	−0.3 (5)
N15^iii^—K2—N8—C14	−165.3 (3)	Pd2—N7—C14—N8	178.8 (3)
N15^vi^—K2—N8—C14	−86.3 (3)	C11—N7—C14—C15	178.2 (3)
N13—K2—N8—C14	−43.3 (4)	Pd2—N7—C14—C15	−2.8 (4)
N9—K2—N8—C14	3.4 (3)	C16—N9—C15—N10	−0.1 (5)
N12^iv^—K2—N8—C14	126.6 (3)	K2—N9—C15—N10	−172.9 (2)
C19—K2—N8—C14	61.7 (3)	C16—N9—C15—C14	179.8 (3)
S8—K2—N8—C14	175.7 (2)	K2—N9—C15—C14	7.1 (4)
K2^vii^—K2—N8—C14	−126.1 (3)	C18—N10—C15—N9	2.4 (5)
N11—K2—N8—C13	−120.3 (3)	Pd2—N10—C15—N9	−173.7 (3)
N15^iii^—K2—N8—C13	11.2 (3)	C18—N10—C15—C14	−177.6 (3)
N15^vi^—K2—N8—C13	90.2 (3)	Pd2—N10—C15—C14	6.4 (4)
N13—K2—N8—C13	133.2 (3)	N8—C14—C15—N9	−3.8 (5)
N9—K2—N8—C13	180.0 (3)	N7—C14—C15—N9	177.7 (3)
N12^iv^—K2—N8—C13	−56.8 (3)	N8—C14—C15—N10	176.1 (3)
C19—K2—N8—C13	−121.8 (3)	N7—C14—C15—N10	−2.4 (4)
S8—K2—N8—C13	−7.7 (3)	C15—N9—C16—C17	−2.3 (6)
K2^vii^—K2—N8—C13	50.4 (2)	K2—N9—C16—C17	170.3 (3)
N11—K2—N9—C15	−121.0 (3)	N9—C16—C17—C18	2.3 (7)
N15^iii^—K2—N9—C15	10.0 (3)	C15—N10—C18—C17	−2.3 (6)
N8—K2—N9—C15	−5.3 (2)	Pd2—N10—C18—C17	173.1 (3)
N15^vi^—K2—N9—C15	69.4 (3)	C16—C17—C18—N10	0.1 (6)
N13—K2—N9—C15	151.7 (3)	Pd2^viii^—S3—C19—K2	79.2 (2)
N12^iv^—K2—N9—C15	−55.2 (3)	N15^iii^—K2—C19—N11	106.2 (3)
C19—K2—N9—C15	−107.6 (3)	N8—K2—C19—N11	174.8 (3)
S8—K2—N9—C15	−164.9 (2)	N15^vi^—K2—C19—N11	−135.2 (3)
K2^vii^—K2—N9—C15	44.9 (3)	N13—K2—C19—N11	−36.9 (3)
N11—K2—N9—C16	66.8 (3)	N9—K2—C19—N11	−130.7 (3)
N15^iii^—K2—N9—C16	−162.2 (3)	N12^iv^—K2—C19—N11	109.5 (3)
N8—K2—N9—C16	−177.5 (3)	S8—K2—C19—N11	35.9 (3)
N15^vi^—K2—N9—C16	−102.8 (3)	K2^vii^—K2—C19—N11	158.9 (3)
N13—K2—N9—C16	−20.5 (3)	N11—K2—C19—S3	176.0 (5)
N12^iv^—K2—N9—C16	132.6 (3)	N15^iii^—K2—C19—S3	−77.8 (3)
C19—K2—N9—C16	80.2 (3)	N8—K2—C19—S3	−9.2 (2)
S8—K2—N9—C16	22.9 (5)	N15^vi^—K2—C19—S3	40.8 (3)
K2^vii^—K2—N9—C16	−127.3 (3)	N13—K2—C19—S3	139.1 (2)
N7—Pd2—N10—C18	178.4 (3)	N9—K2—C19—S3	45.3 (2)
S4—Pd2—N10—C18	−1.6 (3)	N12^iv^—K2—C19—S3	−74.5 (2)
N7—Pd2—N10—C15	−6.1 (2)	S8—K2—C19—S3	−148.1 (2)

Symmetry codes: (i) −*x*, *y*−1/2, −*z*−1/2; (ii) −*x*+1, −*y*+1, −*z*; (iii) −*x*, −*y*+2, −*z*; (iv) *x*, *y*+1, *z*; (v) −*x*+1, −*y*+2, −*z*; (vi) *x*, *y*−1, *z*; (vii) −*x*, −*y*+1, −*z*; (viii) −*x*, *y*+1/2, −*z*−1/2.

### Hydrogen-bond geometry (Å, º)

**Table:**

*D*—H···*A*	*D*—H	H···*A*	*D*···*A*	*D*—H···*A*
C1—H1···S1	0.95	2.77	3.334 (4)	119
C1—H1···N11^ii^	0.95	2.45	3.134 (5)	128
C6—H6···S7^vi^	0.95	2.76	3.457 (4)	131
C8—H8···S2	0.95	2.77	3.321 (4)	118
C11—H11···S3^i^	0.95	2.82	3.371 (4)	118
C11—H11···N6^vii^	0.95	2.63	3.315 (5)	130
C13—H13···N15^iii^	0.95	2.61	3.376 (5)	138
C16—H16···S6^ii^	0.95	2.83	3.640 (4)	144
C18—H18···S4	0.95	2.71	3.276 (4)	119

Symmetry codes: (i) −*x*, *y*−1/2, −*z*−1/2; (ii) −*x*+1, −*y*+1, −*z*; (iii) −*x*, −*y*+2, −*z*; (vi) *x*, *y*−1, *z*; (vii) −*x*, −*y*+1, −*z*.
